# Parental intuition: a phenomenological structure of intuitive knowing in the context of child illness and shared decision-making in healthcare

**DOI:** 10.1080/17482631.2025.2491925

**Published:** 2025-04-15

**Authors:** Rachel L. Shaw, Gemma Heath, Virginia Eatough, Lisa Thackeray

**Affiliations:** a Psychology and Health Psychologist, Institute of Health & Neurodevelopment, College of Health & Life Sciences, Aston University, Birmingham, UK; b Psychologist, School of Psychological Sciences, Birkbeck, University of London, London, UK; c Child Attachment & Psychological Therapies Research Unit, Anna Freud & University College London (UCL), London, UK

**Keywords:** Intuition, phronesis, parenting, shared decision-making, qualitative research

## Abstract

**Purpose:**

Parents describe knowing instinctively when there is something wrong with their child, but they experience challenges convincing healthcare professionals of these concerns, which could prohibit timely escalation of care. Our purpose was to develop a phenomenological description of parental intuition from parents’ lived experience.

**Methods:**

We interviewed 12 parents remotely using a semi-structured schedule. Interviews were analysed using descriptive phenomenology.

**Results:**

We developed a phenomenological description of parental intuition with essential elements including: parental intuition as pre-reflective and pre-linguistic, as corporeal, affective, instinctive, hysteria, and phronesis. Parental intuition was expressed as prior to consciousness and felt within the body. It manifests as heightened arousal and emotion. Parental intuition was defined as ever-present, yet questionable, potentially gendered, requiring validation. Finally, parental intuition was defined as practical wisdom built up over years of exposure to one’s child, enabling a reciprocal, unspoken and intimate bond.

**Conclusions:**

Our work has demonstrated the significance of parental intuition in early detection of health deterioration. We discuss philosophical conceptualizations of knowledge and evidence relating to healthcare professionals’ resistance to accept parental intuition as a valid source of knowledge in healthcare. We argue that parental intuition demands integration into practice guidance on paediatric shared decision-making.

## Introduction

Clinical intuition is accepted as expert knowledge (Sackett et al., 1996). It is practised as phronesis – practical wisdom – and informs shared decision-making (Ellet, 2012). Shared decision-making involves respectful dialogue between patients and clinicians (Walseth & Schei, 2011). However, this doesn’t translate to paediatrics; parents’ contributions – their intuitions – about their children are not so easily accepted (Paciotti et al., 2014). Instead, parental intuition evokes the concepts of “life instincts” (Freud, 1920), “maternal instinct” (Miller, 2005; Letherby, 1994), mysticism (Michaud, 1998; Rosanoff, 1999); it raises questions about human capability and the stability of knowledge.

This study explored parents’ experiential accounts to develop a phenomenological description of parental intuition. Research has shown when their child’s health deteriorates, parents experience an “instinct”, feelings that “there’s something not quite right” which often are not “heard” (parents; Heath et al., 2016, p. 4; Ray et al., 2009). Clinicians are aware of this but struggle to determine whether parents are “just stressed because their kid has been admitted to hospital or are they genuinely concerned that their child is clinically deteriorating” (nurse; Heath et al., 2016). Parent triggered rapid response and early warning systems have been developed (Dean et al., 2008; McCabe et al., 2009) but work evaluating their efficacy is lacking (Vorwerk & King, 2016). There is little explanation for this, except that while clinicians accept the value of parents’ perspectives, they remain reticent about implementing treatment changes based on them (Paciotti et al., 2014).

Nevertheless, shared decision-making is firmly rooted within healthcare vernacular (Elwyn et al., 2001; :National Co-ordinating Centre for NHS Service Delivery and Organisation R&D, 2005; Wirtz et al., 2006) and evidence of its effectiveness is growing (Coulter & Collins, 2011). Resistance to its adoption comes in perceptions that it takes too long, patients will choose more expensive treatment, low health literary precludes its benefits (Legare et al., 2008). Yet, evidence shows it can save time (Bekker et al., 2004) and disadvantaged groups have the most to gain (King et al., 2011; O’Connor et al., 2009).

Research has failed to ask *why* resistance occurs. We argue this lies within our perceptions of what constitutes valid knowledge and expertise. With knowledge comes power, and by extension, the “permission” to act with authority (Foucault, 1976). Paterson’s et al. (1957) Aesculapian authority has long been used to describe the unique authority embodied by doctors (and arguably by extension, nurses and allied health professionals) (Paterson et al., 1957; Rollin, 2002; Stanton, 1999). Aesculapian authority combines sapiential (or expert) authority, which comes from having more knowledge and expertise than others, and moral authority, which entitles an individual to control and direct others: “What the doctor is doing is socially right as well as individually good. This is an unbeatable combination. There is no other profession that matches this” (Paterson et al., 1957, cited by; Siegler & Osmond, 1973, p. 42). A known threat to Aesculapian authority came in “Doctor Google”, enabling patients to self-diagnose and self-treat thereby rejecting expert authority (Donnelly et al., 2008). Alongside changes in how patients consume medical information, systemic developments in medical and healthcare services have begun to breakdown the historic reverence attributed to the moral power of doctors. Budget cuts and staff shortages observed in the UK and US, for example, have led to the creation of new roles (physician associate, nurse and pharmacy prescribers) which take on some of the medic’s duties without their specialist training and professional social standing (American Association of Colleges of Nursing, 2021; Royal College of Nursing, 2022). Perhaps parental intuition is perceived as a similar threat to the moral authority of the medical profession.

The research questions we asked were as follows: in what ways have parents experienced intuition in the care of their child; and how do parents define parental intuition? Through this question, we challenge the status quo of unquestioning acceptance of the moral authority of doctors to the detriment of other forms of knowing. Our analysis of parental intuition offers a philosophical rebuttal to Aesculapian authority.

## Methodology

A descriptive phenomenological approach was taken using Giorgi’s (2009) method. This sits within Husserl’s (1983) tradition of phenomenology which is committed to understanding how the world, events and people appear to us in our consciousness. Giorgi (1970) was inspired by Dilthey (1996) and others to frame psychology as a phenomenologically sensitive human science that aims to understand the subjective meanings attributed to lived experience in comparison to the natural sciences which were occupied by making predictions and providing explanations for natural phenomena (Moran, 2000; Palmer, 1969).

Ethical approval was provided by the host university (ref: LHS1128) to recruit participants through professional networks using social media, professional connections, and UK forums: www.mums.net and www.netmums. Inclusion criteria were that individuals were parenting at least one child currently living with them. We used “parent” to refer to a mother or father as defined in law, i.e., through birth, adoption or in another legally binding role, e.g., foster carer or guardian.

Volunteers were sent a Participant Information Sheet, given an opportunity to ask questions of the research team, and provided their informed consent before continuing. Semi-structured individual interviews were conducted. Participants were asked to describe themselves and their family. Then, we asked them to think of a specific situation when they experienced a feeling that “something was not right” with their child. We probed further detail, asking them to name and describe that feeling (see Table 1). Participants were given a pseudonym, and any identifying information was removed during transcription. All data were stored securely on the host university’s GDPR-compliant cloud storage, only accessible by password by the research team. Recordings of interviews were made on encrypted voice recorders and/or video conferencing software. Recordings were downloaded to the secure cloud storage and deleted from voice recorders and video conferencing software.

**Table 1.** Interview schedule.

Preamble	
We are interested in understanding more about those times when parents think and feel there is something wrong with their child or when they have concerns about their child’s health and wellbeing. We are interested in understanding this experience from your perspective including things such as, how you knew something was wrong, who you talked to and turned to for support and how easy or difficult it was to get help. There are two main topics we would like to ask you about: • describing times or situations when you knew or felt something was wrong with your child and what happened; • helping us to understand the role of this specific kind of knowing within your parenting practice.	
Gaining contextual information	
*Exploratory question*	*Probes for further detail*
Can you tell me something about X (child)? (Aim to build rapport, get a sense of the child, elicit other contextual information).	• What does he/she like to do? • Friendships and so on …
Section 1: Focusing explicitly on concrete situations	
Begin with an initial situation where the parent knew there was something wrong and aim to get a rich description. Then ask for other times they had this experience, dealing with each one separately and in detail. Let the parent introduce the times that are significant to them.	
Q1. Think about a specific time or situation when you experienced the feeling that something was not right with your child.	• Can you describe it? • Where were you? • What was the situation? • What were you doing? • Who was there? • How did you know? • What were the signs? • What did you feel? • Where in your body did you feel it? • What did you think? • What did you do about it?
Q2. This sense of knowing and feeling when something is wrong with your child, what do you call it?	• How would you describe it?
Q3. Can you tell me about who you first told about X (the name they use) and your concerns? (Prompts—if they mention healthcare professionals follow up with family/friends or vice versa).	• How did you communicate/express it to others? • What was the response? • What was the outcome? • How did you feel?
Q4. Can you think of a time when you’ve felt that something was wrong with your child, but you did not act on it?	• Can you describe it? • What happened? • What prevented you from acting
Q5. Can you think of a time when your X (feeling) has been proved wrong?	• Can you describe it?
Section 2: Understanding the phenomenon	
Q6. What role do you think X plays in your parenting?	
Q7. How important is X (using the name they use) for you in terms of being a parent?	
Q8. How important is X in terms of helping you to know when you need health professionals to take your concerns seriously?	
Q9. Where do you think X comes from? (e.g., innate, learnt, experience)	
Close	
Q10. Is there anything more you would like to add?	

Twelve participants were recruited from mixed backgrounds (see Table 2). Their children’s ages ranged from just over one year to mid-thirties. Interviews were conducted in 2016, 2017, and 2020 in person or via video call. Interviews lasted from 30 to 90 minutes, with the majority being around one hour.

**Table 2.** Participant details.

Pseudonym	Age	Ethnicity	Occupation	Partner’s pseudonym	Child pseudonyms	Child ages
Adam	40–45	South Asian Bangladeshi	Counsellor	Beatrice	Crystal Daniel Ellen Fiona	23 21 17 11
Alice	30–35	White British	Psychologist	Chris	Belinda	3
Bella	40–44	South Asian	Counsellor	Tom	Sam	7
David	60–64	White British	Nurse	Evelyn	Lee Michael Neil	36 33 25
Francis	35–40	White British	Child Protection Officer	Gary	Robert Sarah	12 7
Hope	35–40	Zimbabwean	Nurse	Isaac	Joanna Kevin	18 14
Isabelle	35–40	South Asian	IT Project Manager	Jack	Carl Dominic	4 1
Juliette	35–40	White British	Police Officer	Kenton	Laura Maria	3 2
Lauren	45–50	White British	Podiatrist	Matthew	Eli Freddie	16 19
Lennox	40–44	African-Caribbean	Healthcare Assistant	Mandy	Paul Stuart Tania	15 13 10
Nora	35–40	South Asian Pakistani	School Caterer	Oliver	Lily Miranda Noah	17 15 9
Veronica	30–35	White British	Police Officer	William	Alexander Benjamin	5 4

Descriptive phenomenology was used to produce a structural description of parental intuition (Giorgi, 2009). The first phase was to read transcripts closely and dwell in each participant’s account. Next, transcripts were divided into meaning units. Summaries were made against meaning units and simultaneously, their psychological significance was noted. Individual descriptions of the phenomenon were then developed. The final phase involved a shift from idiography to the transcendental. Lists of candidate constituent elements from each individual account were synthesized and we engaged in the eidetic reduction. This meant each potential element was tested against all participants’ transcripts through imaginative variation; questions were asked of each element in our description, and whether if it was taken away, the phenomenon of parental intuition remained feasible. Imaginative variation did result in a changed structure as elements were interrogated and abandoned, morphed into something else, or retained. The use of imagination is significant because it enables us to escape the confines of the natural attitude, to challenge what is taken for granted, and imagine multiple variations of reality which help us to grasp the essential meanings of a phenomenon.

## Results

Participants identified episodes in their children’s lives which were accompanied by an experience of parental intuition. The different forms of parental intuition articulated represent a kaleidoscope of experiences, moveable lenses that can be adjusted to bring into focus the essential elements -or phenomenological structure – of parental intuition (see Table 3). It is important not to read the themes as objective fact, but as the product of a meaning-making process that represents parents’ experiences of the phenomenon.

**Table 3.** The Phenomenological Structure of Parental Intuition.

Parental intuition is pre-linguistic, pre-reflective. It is corporeal and affective. Parental intuition conveys a signal of something only discernible by the parent. Parental intuition is built on a lifetime’s exposure to your child. It is experienced as instinctive, natural and cannot be ignored. Parental intuition is felt in the gut, it is an increased heart rate, it is your heart dropping to your belly. Parental intuition feels like a stress response, it is panicky, a blinding flash. Parents are uncertain about what qualifies as parental intuition and fear that if conveyed others will perceive them as daft, stupid, hysterical, a crazy parent. Parental intuition is not always right and is not experienced by all parents. It is challenging to convince others of the authenticity of parental intuition. Parental intuition acts as a hypothesis to be tested by gathering evidence that corroborates (or rejects) it. Professional intuition has similar constituents but is ratified in the rational realm of techne. Parental intuition is akin to phronesis and draws on a different kind of knowing.

To further examine this phenomenological description, each essential element is presented alongside data extracts (see Table 4).

**Table 4.** Essential elements of parental intuition.

Essential elements	Description
*Parental intuition as pre-reflective and pre-linguistic*	Something occurring prior to full consciousness, before being put into words
*Parental intuition as corporeal*	A felt sense, experienced in the body
*Parental intuition as affective*	An emotional response or heightened sense of physical arousal
*Parental intuition as instinctive*	Ever-present, yet somehow inexplicable
*Parental intuition as hysteria*	A questionable, (probably) gendered, kind of knowing which requires validation
*Parental intuition as phronesis*	A practical wisdom built up through years of exposure to one’s child, an established reciprocity, a bond that enables an intimate form of knowing exclusive to parents (or primary carers)

### Parental intuition as pre-reflective and pre-linguistic

When asked to identify a time when “something was not right” with their child many struggled to articulate it beyond an initial, equivocal description: “a fear that something is wrong” (Hope); “a parent’s intuition [is that] you know when something is wrong” (Adam). Others explicitly stated their difficulty naming it: “that feeling that you get that, you can’t put into one word” (Lennox); “I can’t give it a name” (Nora). The feeling of parental intuition and the knowledge it generates are familiar, yet it remains obscure.

Alice’s story of when Belinda, aged three, dislocated her elbow illustrates this pre-reflective element of parental intuition. Alice and family were staying in an old country house for their vacation. The family returned to the house after a long walk and were getting ready for the evening’s activities when Belinda started “throwing an absolute, awful tantrum”:

She kept running off and climbing these stairs and so I was trying to get her to come back. It had been a really long day, and she decided she was just going to scream and scream and scream. It was awful. My husband was just off chatting to people and I was there holding umpteen bags and a screaming child who I couldn’t get to move down this corridor. She kept dropping to the floor and dropping to the floor. Then she became very, very upset. And it was different to the tantrum upset and I thought something’s not quite right, and I thought, maybe she’s just got beyond distressed, so I scooped her up and put her to bed, but she wouldn’t settle at all. It turns out she’d got a dislocated elbow, from dropping and pulling and dropping down to the floor so much, which is apparently very common. So there was me thinking I’m a fail, I’ve injured my child! (Alice)

In this moment, Alice experienced many emotions; she was tired, over-burdened, alone, trying to appease a distressed toddler. Despite this, Alice could read a significant shift from “tantrum” to something else:

I could just tell by her demeanour and the way she changed. It was a different type of crying. I can’t really explain it, but it went from being stroppy crying to being, she was so distressed [.] so we ended up in A&E. [Accident and Emergency Department/Emergency Room] (Alice)

When pressed to elaborate, Alice too found it difficult to articulate. It was a felt sense, a sign she had perceived in her child’s cry:

Interviewer: know you said it’s difficult to explain, but, can you describe a bit more about that time when you thought, “this is not right”?//

Alice: //this is not right. The first, so, as I started to think this isn’t, there’s something wrong, I just started to get more worried about it, because she’d never been, she’s never done that, she’d never been like that before [long pause] I guess she was, she was just in pain and didn’t, it must have really hurt. [.] so I went and got my husband and I was like, I think there’s something not right here, and I think she’s hurt herself and you start trying to make sense of what could this problem be. And then she started to move and she was like “ouch, ouch!” and it’s like, okay, you’re trying to find which bit hurts and you’re poking, “does this hurt?”, kind of thing.

This is Alice’s conscious attempt to put language to her pre-reflective sense that there was something wrong. Alice had intuited this from a changed tone in Belinda’s cry. Later, she tries to unpick further how she knew and challenges whether this was intuition:

It wasn’t that it was obvious signs of pain and she was very distressed so I don’t think it was intuition, it was just being aware of what was in front of me [long pause] I just think it was obvious to me, but then it may not have been obvious to someone else [.] I knew before Chris [husband] was there and he didn’t really pick up on it, but I don’t think he was paying much attention to what was going on. [.] But I don’t think it’s intuition, I just think it’s spotting, it’s knowing signs, it’s knowing how they respond [.] I don’t know why I’m comparing myself to Chris, but he doesn’t. (Alice)

Alice’s struggle to discern whether this was intuition illustrates its ephemeral nature. Nevertheless, her description of a heightened sense of attunement to her child and her ability to “receive” signals Belinda conveyed, suggests a pre-reflective connection not shared by others. Alice went on to explore whether different signs might only be discernible through intuition. She described the emotionality of children in their younger years and their quick shifts in mood:

They’re very emotional aren’t they? I think that makes it hard to figure out what is going on for them. She’ll go from being absolutely happy to utterly miserable in an instant and you think what has happened, and I quite often say to her, “what happened in the last 30 seconds that has caused this sadness?”. That’s when there might be something wrong or she’s just having a moment, so maybe that’s when intuition comes in, when you, it’s not a moment, it’s actually that there’s something wrong. (Alice)

Alice proposes that reading these emotional signs equates more closely with her understanding of parental intuition than the perception of pain in her earlier story. Perhaps this is related to the “evidence” available to confirm the intuitive knowing. The pain of the dislocated elbow was “confirmed” by diagnosis in hospital. This tangible, bodily break, fits well within the technical realm and therefore perhaps does not require the “softer” intuitive sense of knowing. But, is Alice doing herself a disservice here, because she did perceive that change in demeanour when others didn’t. Alice then implies that parental intuition is contingent upon it being “proved right”. In the later example, the contingency upon proof remains, but there is also a suggestion that intuition is discernible only through the parental connection rather than a medical diagnosis. For Alice, then, intuitive knowing is different from medical knowing. However, she is reticent about labelling the ability to perceive something hidden from others as parental intuition. Nevertheless, she is willing to conceive the recognition of a change in affect as intuitive knowing that is only accessible to parents.

### Parental intuition as corporeal

Several participants initially referred to parental intuition as a “gut feeling” (Veronica). For others it was experienced as a heightened state of physical arousal: “I got quite anxious” (Alice); “I was, I wouldn’t say panicked, maybe I was a bit panicked” (Lauren); “that alarm bell inside my head” (Nora). For Veronica, this is experienced as a strong emotional and embodied feeling:

Veronica: It’s just like, I know like, he’s my child, I know there is potentially something there [.] it’s just like a gut feeling, like sick, like, it’s really hard to describe, and it’s quite an emotional, strong feeling.

Interviewer: Does it make you feel kind of like, I don’t know, tense, ‘cause you did that with your fist [makes a fist]?

Veronica: Yeah, it’s like a fit of frustration as well [.] I just feel sick, in that I know that he struggles with some things [.] makes me question myself a little bit, because it’s like [sighs] am I just imagining it? Because nobody’s listening to me.

The frustration Veronica feels is related to a felt need for validation. Despite this powerful felt sense that her son, Alexander, is showing signs of autism, Veronica is not taken seriously by health professionals. This lack of recognition leads to self-doubt which fundamentally undermines her belief in her intuition. Others described being “fobbed off” (Bella) or “not being taken seriously” (Nora), which similarly worked to threaten the validity of parental intuition as a felt bodily sense.

Francis, though, articulated her embodied intuition vividly:

Interviewer: When you said you felt like a wave of panic came over, can you just describe that a bit more?

Francis: Yeah, I can describe it perfectly. Tingling on the top of my head then it feels like hot liquid literally going down the back of my head, and then I feel like the left side of my neck, here, starts to tingle, like really severe pins and needles and my voice goes deeper, like it’s muted [laughs]

Interviewer: So a real sort of, a sense of panic?

Francis: Yeah and like my hearing changes so outside stuff would, I’d have like a high pitched ringing in my ear so talking to you now, the hearing, I wouldn’t be able to hear you properly, but if I was to touch the side of my neck or scratch my finger or move my hair, that sound would be totally amplified [.] I do get a lot of physical warnings I suppose or reactions.

For Francis, intuition is an embodied response, signalled by overwhelming physical sensations. The physicality of her intuition focuses her senses inward, making it difficult to perceive anything else. When asked if she acts upon these physical warnings, Francis says, “Oh God yeah, yeah, yeah definitely, I always act on them!”. There is certainty in Francis’ account not found in others. This may be related to the corporeality of Francis’ intuition; physicality offers itself as evidence.

### Parental intuition as instinctive yet skilful

Juliette also displays certainty because she has spent years training to use her intuition professionally. Juliette is a police dog handler. Juliette provided behavioural training to aggressive dogs and described how she used that skillset in her parenting:

With dogs it’s all in the body language so I had got quite a sharp eye on reading a dog. It sounds silly but I do really think that’s really trained me with my children. (Juliette)

The reading of non-verbal cues required to train animals aided Juliette’s parenting. She was trained to be observant and has carried this through to her parenting. Like others, Juliette describes watching her children “all the time”:

I watch them, even the subtle signs, you know, I do feel comfortable doing it, it’s [dog training] my first learning of being in charge of something or having the responsibility for something is looking and watching [.] I can watch my child and I can read them very well and I can anticipate [.] In my job I can see when it’s gonna erupt way before the eruption, you can just see the trigger points [.] I definitely feel confident in doing that. (Juliette)

This professional intuition was further embedded for Juliette through her training of others. She debated whether this intuitive knowing is innate or something which can be taught:

There may be a bit of natural ability, I don’t know, I wouldn’t like to say if I’m honest, but, through life experiences of knowing what to look for- but it was definitely a learnt behaviour, I don’t think it’s instinctive, I don’t know, I just, I naturally do it now, it’s just instinc- it’s just in me. (Juliette)

There is reticence to use “instinctive” here, but that feeling of “it’s just in me” suggests innateness. There is also a sense that Juliette’s intuition *feels* instinctive because she has spent so many years perfecting it. The technicality of professional learning provides some rational buffering to the reticence expressed towards owning an instinct.

### Parental intuition as hysteria and hypervigilance

Translating that pre-reflective felt sense into words was experienced as challenging. Participants described inward struggles in naming and owning their intuition which were magnified when conveying its significance to a third party. In our increasingly technologized healthcare contexts this is particularly challenging. Lauren describes her lengthy experience of getting a diagnosis for her son, Eli:

Both of my children are very tall and they were very slim, particularly as young teenage boys, they became very tall and very slim very quickly and I remember worrying about my eldest child for the same reason and then the youngest one as well because he had suddenly become quite thin, but I put it down to normal growth patterns, which potentially it was, but when he was thirteen and a half we went on a long walk, a coastal, hilly, quite demanding walk, and he had an episode where he almost collapsed, went white as a sheet, had chest pains, was quite sick, you know, to be honest, kind of the symptoms of a heart attack [laughs] but obviously it wasn’t, but clearly there was something very not right. (Lauren)

In a remote location, away from emergency services, this was a distressing experience. Although Eli recovered that day, Lauren knew “objectively those symptoms in a child that age are not normal”. When they returned Lauren consulted her general practitioner (GP), suggesting her son was anaemic. This idea was “poo-pooed”, but tests were carried out and Eli was indeed anaemic. Now prescribed iron tablets, Eli’s fatigue improved, but he continued to be ill, lost more weight, and stopped eating properly. Lauren then engaged in an exercise of differential diagnosis. Eli continued to be “symptomatic” following treatment for anaemia. Lauren became increasingly worried and began working through options:

Still people were just going, “oh he’s just thin”, you know the GP I went to with the stomach pain initially, it was either the first or second time I went in I said, “can you check, we have a lot of autoimmune disease in our family [pause] lots of it”, and I said, “do you think this is celiac disease?”, “I don’t know, I’ll check it”, “do you think this is Crohn’s disease?”, “oh no, it’s definitely not Crohn’s disease”, “oh okay” [.] but at that point you sort of accept, you bow to their superior knowledge don’t you? [.] But I’d reached the conclusion quite early on, but the night sweats, also, I had a bit of a blinding flash, because I realised that it’s also the symptoms of lymphoma and at that point, you know, your heart drops to your stomach really, and I just thought, “oh shit!”, you know, what if it is. And at that point, I thought, “right, we need this investigating now”. (Lauren)

Lauren was now driven by fear. However, she was aware her behaviour may influence healthcare professionals’ response to her concern and was very cautious to present as “rational”. In this struggle, Lauren was straddling the positions of parent of a sick child and a healthcare professional herself. Lauren drew upon this professional experience to help navigate the system:

I was trying to be very rational, I was trying very hard, just to be rational about it, because I just thought me being hysterical is not gonna help the situation really. It’s helpful that I’m used to dealing with medical professionals and it’s helpful to be able to go and say, “actually this is what I want you to do,” which is what I did in the end. I went back to the GP probably the third time, and said, “right, I want you to refer him to a paediatrician now please”, which they did, I think probably because I was assertive about it. He was seen at our local hospital and the paediatrician again [pause] I could tell by the way she was talking to me just felt I was overreacting and there was actually nothing wrong with him and he was just a bit thin. [.] There’s a stool faecal test they can do which shows a protein which is only present in bowel cancer and Crohn’s and it was absolutely through the roof! [pause] At that point, they realised what was wrong with him. (Lauren)

Trying to convince healthcare professionals that her intuition was legitimate took immense strength of will, persistence, and rationality, but it was insufficient. It took “technical” evidence of the medical test to achieve a diagnosis of Crohn’s disease.

Francis’ experience was similar. She struggled getting an asthma diagnosis for her daughter, Sarah. Sarah’s journey involved many repeats consultations and visits to the Emergency Department. On one occasion, Francis knew “there was something else and no one was listening to me at all”. Francis described the feeling as nurses tried to placate and reassure her that the machines would detect if there was something wrong:

Francis: No [pause] there’s something not right.

Interviewers: And how did you know that?

Francis: My stomach literally, you know when you gotta do an exam, a big exam, you feel nervous, and I think for girls it’s often felt really low down, like the adrenaline like here [pointing to her lower belly, her womb?] and my stomach was just in knots, like I was totally flooded with adrenaline, I was sweating, but I was cold, my heart rate was going, dry mouth, and I just felt in a complete panic [.] I remember thinking, “this is such an injustice, no one will believe me, how can I get people to believe me, there’s something extra wrong with Sarah, there’s something really wrong?” [.] so at this point I’m starting to go a little bit round the twist here, and I was also worried about sabotaging the relationships that I’d built up with the nurses and doctors ‘cause they’ve always been so kind to us [.] I was trying to reason with myself thinking is it worth [pause] sabotaging these relationships by kicking off and going mental at her? [.] you start doubting yourself [.] I just sat there [.] I must have looked crazed because of the amount of adrenalin pumping round my body, I was shaking, my hands were shaking, I couldn’t eat, my voice was croaky, but I just thought, “there’s sod all I can do about it, no one’s gonna listen to me” [.] I don’t want to be one of those mums that kicks off.

Francis makes clear it is not simply her child’s health at risk, but also her parental credibility. Francis’ experience had shown a good rapport with healthcare professionals was essential. Yet, when at breaking point, she was willing to sacrifice her rationality and sabotage those relationships to save her child’s life. Francis’ suspicion was proved right as Sarah went into respiratory arrest and rapid response lifesaving treatment was required, which saved her. There was a greater cost for Francis, though. She had developed post-traumatic stress disorder (PTSD) following a separate incident some years previously. After this episode with Sarah, she experienced PTSD symptoms and became “hypervigilant” in Sarah’s care.

In a similar way, Alice described how “I always watch the kids” to make sure they’re okay, especially after being away from them and when they have been in someone else’s care:

Whenever I come in from work, I always watch the kids for a little while. I’ll always go in and say, “ah hello, how are you? Blah blah blah” but I’ll always watch them for a little while just to check, I don’t know what I’m checking for, that’s interesting, I don’t know, but I’m checking. (Alice)

Rationally, Alice knows her children are well cared for while she is at work, but something inside requires her to check. Perhaps this (hyper-)vigilance is what enables parents to develop intuition. The many hours spent with a child, intimate knowledge of their body, moods, mannerisms create an intricate tapestry, revealed only to parents. Others may be able to make out elements of the image, but parents alone are able to see the whole picture.

### Parental intuition as phronesis

The notion of parental intuition as “practice” built up over time suggests a connection to phronesis—practical wisdom that enables individuals to discern what is the right action to take right now, in this context (Aristotle, transl., 1976, cited by; Kinsella & Pitman, 2012).

To illustrate this phronesis -parental expertise- we turn in more detail to Francis’ daughter, Sarah. As an infant, Sarah had experienced respiratory problems, the first episode started with a cold during her first Christmas:

She was ill all over Christmas and I’d not seen her ill before. She looked so ill and purple, purple eyes, and we kept going in and out of the doctors and they she had respiratory bronchitis or something. He gave her steroids, and she’s a tiny baby, we had to scrunch it up and throw it down her throat sort of thing. I just knew, there’s something really wrong with my baby. [.] I thought [exhales] looking at her, my baby’s not well, I know what a sick baby looks like. This is more than a cold or aggravated bronchitis [.] and she just didn’t get any better. (Francis)

Despite Sarah being newborn, Francis expresses with certainty she knew there was “something really wrong”. She asserts this confident knowledge through her prior experience of parenting a sick child. The question is whether this is based on intuition. Like Alice, Francis describes signs she perceives from her baby, this time visual; she “just knew” from looking at her that she was far sicker than her doctor suggested:

All through that year, every three weeks pretty much, Sarah would be rushed into hospital, and I would ring the doctors and say, “my baby’s not very well” because she’d always present with the same symptoms and I got so sick and tired of repeating myself, like I could literally reel it off, just “blah, blah, blah, blah, blah!”, knowing it off by heart, “cause I was just in hospital all the time. I’d start with the doctors [.] ‘what’s the matter?’, ‘well to me it looks like she’s struggling to breathe, she’s lethargic, her temperature spikes at 38 to 39, she’s no energy’. [.] We’d start with the doctors, they would say it’s a virus”, send me home again [.] It’s always worse at night isn’t it so in the middle of the night it would often be an ambulance to the hospital. (Francis)

Francis was frustrated with the healthcare professionals’ unwillingness to accept her account of signs of Sarah’s illness. The need to validate that intuition with additional evidence emerged. Sarah offered the doctor a rehearsed spiel in a futile attempt to be taken seriously. The only avenue open to her is to take matters into her own hands and take Sarah into hospital.

Rather than feeling a need to validate her intuition or being reluctant to own it, Francis’ intuition is secure, but the struggle comes when conveying it. Her judgement as a parent doesn’t convince the healthcare professionals that Sarah’s condition is life threatening. Nevertheless, Francis’ intuition was “proven” in Sarah’s treatment: she was put on a nebulizer, her breathing returned to normal and she recovered. Despite their dismissal of Francis’ intuition, she was co-opted into caring for Sarah while she was hospitalized:

She would have drips in either arms, they’d be pumping her with steroids and hydrocortisone and various other bits and bobs. Then I’d have to do the salbutamol every hour, the ten puffs, I had to train Sarah to accept having this thing go over her face, and they were treating her as if she had asthma, but they refused to diagnose her with asthma, which made me really cross because, I was like, I know my child, I can tell you the day before we end up in hospital, I know that we’re gonna get to this stage because I can see it in my child, you know, her eyes go purple, her neck, there’s this part here [pointing to her neck] would go in, but the very subtle signs were there earlier, y-y-y-ou can think, when you know you spend that time with your child, bearing in mind I was on maternity leave then, I would notice the slightest little change in her personality, you know, not being as active, and I knew then that another episode’s on the way [.] the eyes go purple, the change in personality, you know, she’d withdraw, she’d start to tummy breathe … (Francis)

Through her ability to make sense of signs Sarah displayed, and her practised care of Sarah, Francis became an expert. From that initial intuition that was something was seriously wrong, Francis was able to recognize early warning signs – changes in her baby’s colour, her breathing – and could predict an “episode”. By Alice’s criterion of being proved right, this would “qualify” as parental intuition.

## Discussion

This phenomenological analysis has produced the first structural definition of parental intuition. The value of this work is it clearly portrays parents’ challenge to be heard by healthcare professionals, while simultaneously, demonstrating how crucial their parental intuition is in discerning real threats to their child(ren)’s (quality of) life.

What this analysis doesn’t do is provide an answer to *why* healthcare professionals are reluctant to incorporate parental intuition into their treatment decisions. Aristotle can help unpick this problem. The dominant form of knowledge in contemporary healthcare science is the episteme: scientific, universal, invariable, context-independent knowledge (Kinsella & Pitman, 2012). This chimes well with the hierarchy of knowledge (Sackett et al., 1996) which situates systematic reviews of “gold standard” experiments -randomized controlled trials (RCTs)- at the top of the hierarchy. RCTs use “well-controlled”, nomothetic experimental methods within the positivist paradigm, designed to remove subjectivity to occupy the position as the best quality, “hard evidence”. While this knowledge has significant value in informing healthcare practice, it is not meant to be used in isolation (Shaw et al., 2014). At the heart of evidence-based healthcare, alongside RCT evidence, is clinical expertise, without which, healthcare practice -and consequently- patients would suffer (Sackett et al., 1996).

Clinical expertise could be compared to Aristotle’s intellectual virtue of phronesis. Phronesis is generally defined as practical wisdom which: involves deliberative – but typically not calculative – judgement; complicated interactions between the general and the practical; which manifests as an embodied social practice (Ellet, 2012). In our phenomenological analysis, we have characterized parental intuition as involving phronesis, but it is perhaps more appropriate to conceive of them the other way around – intuition as an active element of phronesis. This chimes with Aristotle’s (1976) notion of intuition as the *process* through which objects in the world are presented to consciousness and deliberate judgements are made through phronesis (Braude, 2013). Furthermore, Gadamer’s and Gadamer (1998) Heideggerian analysis of phronesis is that it brings together technical issues (tacit clues or empirical observations), hermeneutic activity (the process of making sense of perceptions) and the action-oriented outcome (judgement) (Bernasconi, 1989; Heidegger et al., 2003). To develop our philosophical understanding further, Merleau-Ponty’s (2009) concept of habituation helps us conceive of parental intuition as an embodied -corporeal- and relational sense-making process. Habituation emphasizes how a parent’s intuition is built-up over time through accumulated interactions with their child—and that these interactions are embodied: “it is the body which ‘catches’ (*kapiert*) and ‘comprehends’” (Merleau-Ponty, 2009: 165). This dynamic history of parent–child interactions enables parents to attribute meaning to signs conveyed by their child beyond what is immediately observable.

Phronesis, then, acts as counterpart to episteme as knowledge that is pragmatic, variable, context-dependent and oriented towards action (Kinsella & Pitman, 2012). It is clear how these two forms of knowledge complement each other to provide judgements based on “hard” scientific evidence (episteme) *and* the kind of knowledge “earned” by experts, a virtue of experience which might reveal itself as “clinical intuition” (phronesis). When this pairing is joined by Aesculapian authority of healthcare professionals, it not surprising it becomes the most compelling evidence when a life and death decision is required. However, we argue it is time for us to consider other forms of expertise as equitable to that which comes from the prestigious position of doctor.

Surely in paediatrics, it is parents who have unique knowledge affording them the ability to intuit signs from their child not readable by others (Kruithof et al., 2019). It is logical that healthcare professionals create space for parents’ knowledge -parental intuition – especially when there is uncertainty from other sources of evidence (Kruithoff et al., 2019). Certainty of expertise is a socially constructed phenomenon, which arbitrarily closes down routes to knowledge because of historically embedded cultural codes of practice. Benner’s (1984) seminal work demonstrates significant challenge for clinicians in becoming expert and trusting their intuitive judgement. As research has examined clinician expertise (e.g., Paton & Kotzee, 2021; Kinsella & Pitman, 2012; Benner, 1984) we now call for research to further examine parental intuition, the conditions required to produce it and mechanisms by which it can be authentically adopted in clinical practice.

This will require a shift away from contemporary conceptualizations of effectiveness and patient benefit. Family-centred care models offer individuals more “choice and voice” in healthcare decisions, which is a positive move away from paternalistic dogma of the medical model (Dahlberg et al., 2009). Nevertheless, hidden beneath this “empowerment” of patients is a sturdy rationale steeped in politics and economics, not, arguably, an authentic will to improve patients’ quality of life. Family-centred care models operationalized in western countries are economically positioned in measures of cost-effectiveness, they are political because of their rhetorical and vote-winning power and are population-based in prioritization of epidemiology and public health. This population approach is convincingly beneficial, but it moves the focus away from individuals’ lived experience. We argue for a dual approach: alongside the nomothetic population-wide approach – episteme – we need to consider socio-cultural aspects, the multifactorial ways in which healthcare changes across the life-course, and individual agency – lifeworlds – and one’s capacity for action and behaviour (Kelly et al., 2009). This requires an idiographic commitment, acceptance of context-dependent and pragmatic ways of knowing -phronesis. To achieve this, a spectrum of experts is required, each equitable and able to decide what is right and good for the patient.

In the paediatric context, we argue that parents’ unique way of knowing – parental intuition – constitutes fundamental expertise in making the best decisions for children’s health and wellbeing. For the mothers in this study, this type of intuitive knowing may be related to behavioural changes that occur as women transition to motherhood. It is postulated that these changes occur both pre- and postnatally in response to dramatic hormonal fluctuations. These hormonal changes are associated with increases in the size of neurons in some brain regions, and structural changes in others, leading to a focus on offsprings’ survival and wellbeing (Kinsley & Lambert, 2006). Thus, this phenomenological structure of parental intuition demonstrates how this “instinct” manifests meanings that could be operationalized in the development of healthcare plans, treatment pathways, and shared decision-making within multidisciplinary teams.

### Strengths and limitations

This work was undertaken in the UK where patients receive care through the National Health Service, a unique system which differs from many others around the globe. Despite systemic differences however, parallels have been drawn with Swedish healthcare (Dahlberg et al., 2009), which could then be widened further across Europe. The mixed ethnicity of participants and range of stories parents told are real strengths. But of course, there are limitations in that they all lived within a relatively small geographical area in England, UK. A team of interviewers and analysts worked independently and collaborated to produce an agreed structural definition of parental intuition. This piece of work focused on parents, which could be considered a limitation since others clearly have a stake in the phenomenon of parental intuition. Further research with healthcare professionals, partners and siblings would strengthen our understanding of the range of knowledge and expertise required to create a healthcare plan that is right for the patient.

### Practice implications

There is a clear mandate for parents’ knowledge and expertise to be included in decisions about their children’s care in Martha’s rule (NHS, 2025). Martha’s rule is a patient safety initiative which entitles parents (and patients) to an urgent review of care if they believe their loved one’s condition has significantly deteriorated but healthcare professionals are not responding. Delivery models of Martha’s rule are currently being piloted in 143 locations in England with results due in spring 2025. Our research offers a detailed understanding of the beliefs and cultural practices surrounding the pragmatics of healthcare delivery. Alongside an in-depth analysis of the pilot of Martha’s rule, research is required which will: identify whether and how parental intuition contributes to shared decision-making in paediatric care; co-develop interventions to introduce parental expertise into multidisciplinary teams. This research will require involvement from multiple stakeholders, audits of current practice involving parents, coding of parental intuitive judgement processes, and consultation with intervention development theory to identify and test behavioural techniques and intervention strategies that are likely to be effective and sustainable in this context.

## Conclusion

This paper has presented a phenomenological analysis of parental intuition. It used parents’ experiential accounts and philosophical conceptualizations of knowledge to explain why parental intuition is resisted as an established form of expertise within paediatrics. We have produced the first structural description of parental intuition built from empirical evidence. Parental intuition constitutes a form of phronesis that demands integration into paediatric shared decision-making.

## Data Availability

No data can be shared from this project because informed consent was received from participants to do so.

## References

[cit0001] (2005). Concordance, adherence and compliance in medicine taking. NCCSDO. http://www.nets.nihr.ac.uk/__data/assets/pdf_file/0009/64494/FR-08-1412-076.pdf.

[cit0002] American Association of Colleges of Nursing. (2021). The essentials: Core competences for professional nursing education. American Association of Colleges of Nursing. Retrieved February 3, 2025, from http://cnnursing.org/AACN-Essentials.

[cit0003] Aristotle Trans. (1976). *The Nicomachean ethics*. D. Reidel.

[cit0004] Bekker, H. L., Hewison, J., & Thornton, J. G. (2004). Applying decision analysis to facilitate informed decision making about prenatal diagnosis for down syndrome: A randomised controlled trial. *Prenatal Diagnosis*, 24(4), 265–12. 10.1002/pd.851 15065100

[cit0005] Benner, P. (1984). *From novice to expert: Excellence and power in clinical nursing practice*. Addison-Wesley Pub. Co. *compliance in medicine taking: report for the National Co-ordinating Centre for NHS Service*.

[cit0006] Bernasconi, R. (1989). Can phronesis save the life of medical ethics? *Theoretical Medicine*, 17(3), 209–224. 10.1007/BF00489446 8952418

[cit0007] Braude, H. D. (2013). Human all too human reasoning: Comparing clinical and phenomenological intuition. *Journal of Medicine and Philosophy*, 38, 173–189. 10.1093/jmp/jhs057 23339120

[cit0008] Coulter, A., & Collins, A. (2011). *Making shared decision-making a reality: No decision about me, without me*. The King’s Fund.

[cit0009] Dahlberg, K., Todres, L., & Galvin, K. (2009). Lifeworld-led healthcare is more than patient-led care: An existential view of well-being. *Medicine, Health, Care and Philosophy*, 12(3), 265–271. 10.1007/s11019-008-9174-7 19101822

[cit0010] Dean, B. S., Decker, M. J., Hupp, D., Urbach, A. H., Lewis, E., & Benes-Stickle, J. (2008). Condition HELP: A pediatric rapid response team triggered by patients and parents. *Journal for Healthcare Quality*, 30(3), 28–31. 10.1111/j.1945-1474.2008.tb01139.x 18507237

[cit0011] Dilthey, W. (1996). Wilhelm dilthey: Selected works, Volume IV: Hermeneutics and the study of history. In R. Makkereel & F. Rodi (Eds.), *Selected, works IV*. Princeton University Press.

[cit0012] Donnelly, L. S., Shaw, R. L., & van den Akker, O. B. A. (2008). eHealth as a challenge to ‘expert’ power: A focus group study of internet use for health information and management. *Journal of the Royal Society of Medicine*, 101(10), 501–506. 10.1258/jrsm.2008.080156 18840866 PMC2587202

[cit0013] Ellet, F. S. (2012). Practical rationality and a recovery of Aristotle’s ‘phronesis’ for the professions. In E. A. Kinsella & A. Pitman (Eds.), *Phronesis as professional knowledge: Practical wisdom in the professions* (pp. 13–34). Sense Publishers.

[cit0014] Elwyn, G., Edwards, A., Wensing, M., Hibbs, R., Wilkinson, C., & Grol, R. (2001). Shared decision making observed in clinical practice: Visual displays of communication sequence and patterns. *Journal of Evaluation in Clinical Practice*, 7(2), 211–221. 10.1046/j.1365-2753.2001.00286.x 11489045

[cit0015] Foucault, M. (1976 [Trans. 1978]). *The history of sexuality* [Trans. R Hurley]. Pantheon Books.

[cit0016] Freud, S. (1920). Beyond the pleasure principle. In S. Freud, J. Strachey, A. Freud, A. Strachey & A. Tyson (Eds.), *The standard edition of the complete psychological works of Sigmund Freud* (pp. 69–75). Hogarth Press and the Institute of Psycho-analysis.

[cit0017] Gadamer, H.-G. (1998). *Aristoteles, Nikomachische Ethik VI H-G Gadamer (transl)*. Vittorio Klostermann.

[cit0018] Giorgi, A. (1970). *Psychology as a human science: A phenomenologically based approach*. Harper and Row Publishers Inc.

[cit0019] Giorgi, A. (2009). *The descriptive phenomenological method in psychology: A modified Husserlian approach*. Duquesne University Press.

[cit0020] Heath, G., Montgomery, H., Eyre, C., Cummins, C., Pattison, H., & Shaw, R. (2016). Developing a tool to support communication of parental concerns when a child is in hospital. *9 the Global Problem of Insufficient Sleep and Its Serious Public Health Implications Healthcare*, 4(1), 9. 10.3390/healthcare4010009 PMC4934543 27417597

[cit0021] Heidegger, M. (2003). *Plato’s Sophist. R. Rojcewica and A. Schuwer (transl)*. Blookington and Indianapolis: Indiana University Press.

[cit0022] Husserl, E. (1983). Ideas pertaining to a pure phenomenology and to a phenomenological philosophy. *Husserl Studies*. Trans. F. Kersten. The Hague: Martinus Nijhoff (orig. pub. German, 1913.) 1(1), 105–130. 10.1007/BF01569209

[cit0023] Kelly, M. P., Stewart, E., Morgan, A., Killoran, A., Fischer, A., Threlfall, A., & Bonnefoy, J. (2009). A conceptual framework for public health: NICE’s emerging approach. *Public Health*, 123, e14–e20. 10.1016/j.puhe.2008.10.031 1 19100588

[cit0024] King, J. S., Eckman, M. H., & Moulton, B. W. (2011). The potential of shared decision making to reduce health disparities. *Journal of Law, Medicine and Ethics*, 39(S1), 30–33. 10.1111/j.1748-720X.2011.00561.x 21309892

[cit0025] Kinsella, E. A., & Pitman, A. (2012). Engaging phronesis in professional practice and education. In E. A. Kinsella & A. Pitman (Eds.), *Phronesis as professional knowledge: Practical wisdom in the professions* (pp. 1–12). Sense Publishers.

[cit0026] Kinsley, C. H., & Lambert, K. G. (2006). The maternal brain. *Scientific American*, 294(1), 72–79. 10.1038/scientificamerican0106-72 16468436

[cit0027] Kruithoff, K., Willems, D., van Etten-Jamaludin, F., & Olsman, E. (2019). Parents’ knowledge of their child with profound intellectual and multiple disabilities: An interpretive synthesis. *Journal of Applied Research in Intellectual Disabilities*, 1–10. 10.1111/jar.12740 32367663 PMC7687241

[cit0028] Legare, F., Ratte, S., Gravel, K., & Graham, I. D. (2008). Barriers and facilitators to implementing shared decision-making in clinical practice: Update of a systematic review of health professionals’ perceptions. *Patient Education and Counseling*, 73(3), 526–535. 10.1016/j.pec.2008.07.018 18752915

[cit0029] Letherby, G. (1994). Mother or not, mother or what?: Problems of definition and identify. *Women’s studies international forum*, 17(5), 525–532. 10.1016/0277-5395(94)00038-7

[cit0030] McCabe, A., Duncan, H., & Heward, Y. (2009). Paediatric early warning systems: Where do we go from here? *Paediatric Nursing*, 21(1), 14–17. 10.7748/paed.21.1.14.s20 19266776

[cit0031] Merleau-Ponty, M. (2009). *Phenomenology of perception* (Smith, C. (Trans.). Routledge (orig. pub. French, 1945.

[cit0032] Michaud, S. M. (1998). The use of intuition among expert social work practitioners. *Dissertation Abstracts International*, 58(12–A), 4812.

[cit0033] Miller, T. (2005). *Making sense of motherhood: A narrative approach*. Cambridge University Press.

[cit0034] Moran, D. (2000). *Introduction to phenomenology*. Routledge.

[cit0035] NHS. (2025). *Martha’s rule*. Retrieved February 03, 2025, from, https://www.england.nhs.uk/patient-safety/marthas-rule/.

[cit0036] O’Connor, A. M., Bennett, C. L., Stacey, D., Barry, M., Col, N. F., Entwistle, V. A., Fiset, V., Holmes-Rover, M., Khangura, S., Llewellyn-Thomas, H., & Rovner, D. (2009). Decision aids for people facing health treatment or screening decision. *Cochrane Database of Systematic Reviews*, 3, article CD001431. 10.1002/14651858.CD001431.pub2 19588325

[cit0037] Paciotti, B., Roberts, K. E., Tibbetts, K. M., Paine, C., Keren, R., Barg, F. K., Holmes, J. H., & Bonafide, C. P. (2014). Physician attitudes toward family-activated medical emergency teams for hospitalized children. *The Joint Commission Journal on Quality and Patient Safety*, 40(4), 187–192. 10.1016/S1553-7250(14)40024-2 24864527

[cit0038] Palmer, R. E. (1969). *Hermeneutics: Interpretation of theory in Scheiemarcher, Dilthey, Heidegger, and Gadamer*. Northwestern University Press.

[cit0039] Paterson, T. T. (1957). Notes on aesculapian authority. Unpublished manuscript. Cited in M. Seigler & H. Osmond (1973). Values, expertise and responsibility in the life sciences. *The Hastings Center Studies*, 1(2), 41–52. 10.2307/3527512 4802605

[cit0040] Paton, A., & Kotzee, B. (2021). The fundamental role of storytelling and practical wisdom in facilitating the ethics education of junior doctors. *Health: An Interdisciplinary Journal for the Social Study of Health, Illness and Medicine*, 25(4), 417–433. 10.1177/1363459319889102 PMC8276332 31739676

[cit0041] Ray, E. M., Smith, R., Massie, S., Erickson, J., Hanson, C., Harris, B., & Willis, T. S. (2009). Family alert: Implementing direct family activation of a pediatric rapid response team. *The Joint Commission Journal on Quality and Patient Safety*, 35(11), 575–AP6. 10.1016/S1553-7250(09)35078-3 19947334

[cit0042] Rollin, B. E. (2002). The use and abuse of Aesculapian authority in veterinary medicine. *Journal of the American Veterinary Medical Association*, 220(8), 1144–1149. 10.2460/javma.2002.220.1144 11990958

[cit0043] Rosanoff, N. (1999). Intuition comes of age: Workplace applications of intuitive skill for occupational and environmental health nurses. *AAOHN Journal*, 47(4), 156–162. 10.1177/216507999904700403 10418345

[cit0044] Royal College of Nursing. (2022). *Investing patient safety and outcomes: Health and care nursing workforce and supply in England*. Royal College of Nursing. Retrieved Februray 03, 2025, from https://www.rcn.org.uk/Professional-Development/publications/investing-in-patient-safety-and-outcomes-uk-pub-010-567.

[cit0045] Sackett, D. L., Rosenberg, W. M., Gray, J. A., Haynes, R. B., & Richardson, W. S. (1996). Evidence based medicine: What it is and what it isn’t. *BMJ*, 312(7023), 71–72. 10.1136/bmj.312.7023.71 8555924 PMC2349778

[cit0046] Shaw, R. L., Larkin, M., & Flowers, P. (2014). Expanding the evidence within evidence-based healthcare: Thinking about the context, acceptability and feasibility of interventions. *Evidence Based Medicine*, 19(6), 201–203. 10.1136/eb-2014-101791 24799447

[cit0047] Siegler, M., & Osmond, H. (1973). Aesculapian authority. *The Hastings Center Studies*, 1(2), 41–52. 10.2307/3527512 4802605

[cit0048] Stanton, J. A. (1999). Aesculapius: A modern tale. *Journal of American Medical Association*, 281(5), 476–477. 10.1001/jama.281.5.476-JMS0203-4-1 9952212

[cit0049] Vorwerk, J., & King, L. (2016). Consumer participation in early detection of the deteriorating patient and call activation to rapid response systems: A literature review. *Journal of Clinical Nursing*, 25(1–2), 38–52. 10.1111/jocn.12977 26373438

[cit0050] Walseth, L. T., & Schei, E. (2011). Effecting change through dialogue: Habermas’ theory of communicative action as a tool in medical lifestyle interventions. *Medicine, Health Care and Philosophy*, 14(1), 81–90. 10.1007/s11019-010-9260-5 20552281

[cit0051] Wirtz, V., Cribb, A., & Barber, N. (2006). Patient–doctor decision-making about treatment within the consultation—A critical analysis of models. *Social Science and Medicine*, 62(1), 116–124. 10.1016/j.socscimed.2005.05.017 15992980

